# Zirconium–Polycarboxylato Gel Systems as Substrates to Develop Advanced Fluorescence Sensing Devices

**DOI:** 10.3390/gels10120783

**Published:** 2024-11-29

**Authors:** Jon Pascual-Colino, Garikoitz Beobide, Oscar Castillo, Javier Cepeda, Mónica Lanchas, Antonio Luque, Sonia Pérez-Yáñez

**Affiliations:** 1 Department of Organic and Inorganic Chemistry, Faculty of Science and Technology, University of the Basque Country, UPV/EHU, Apartado 644, E-48080 Bilbao, Spain; garikoitz.beobide@ehu.eus (G.B.); antonio.luque@ehu.eus (A.L.); sonia.perez@ehu.eus (S.P.-Y.); 2 BCMaterials, Basque Center for Materials, Applications and Nanostructures, UPV/EHU Science Park, E-48940 Leioa, Spain; 3 Department of Applied Chemistry, Faculty of Chemistry, University of the Basque Country, UPV/EHU, E-20018 Donostia-San Sebastián, Spain; javier.cepeda@ehu.eus; 4 Department of Chemical Engineering, Faculty of Science and Technology, University of the Basque Country, UPV/EHU, Apartado 644, E-48080 Bilbao, Spain; monica.lanchas@ehu.eus

**Keywords:** zirconium, metal–organic gels, fluorescence, chemical sensor

## Abstract

This study presents the development of zirconium polycarboxylate gel systems as substrates for advanced fluorescence sensing devices. Zirconium-based metal–organic gels (MOGs) offer a promising alternative due to the robustness of the Zr–O bond, which provides enhanced chemical stability. In this work, zirconium polycarboxylate gels were synthesized using green solvents in a rapid room temperature method. Fluorescein, naphthalene-2,6-dicarboxylic acid, and 4,4′,4″,4‴-(porphine-5,10,15,20-tetrayl)tetrakisbenzoic acid were incorporated as fluorophores to give the gel luminescent properties, enabling it to be used as a sensor. These fluorophores produce specific changes in the perceived color and intensity of the fluorescence emission upon interaction with different analytes in a solution, allowing a qualitative identification of different solvents and compounds. However, the fragile structure of neat gels hinders reproducible quantitative analysis of fluorescence emission. Therefore, to increase their mechanical stability during manipulation, a composite material was developed by combining the MOGs with quartz microcrystals, which proved to be a more reliable fluorescent system. The results show that the material can identify univocally different solvents and analytes in aqueous solutions by the quantitative analysis of the emission intensities. This work presents an innovative approach to create advanced fluorescence sensors with improved mechanical properties and stability using zirconium polycarboxylate gels and multiple fluorophores.

## 1. Introduction

Metal–organic porous materials have represented a significant research field in materials science during the last decades, with numerous applications [1]. However, in some cases, their stability is inadequate and the material cannot withstand the necessary conditions for the application. To enhance the stability of the system, zirconium-based metal–organic materials can be used due to the strength of the Zr–O bond [2]. Among them, several porous gels (MOGs, metal–organic gels) have been prepared containing [Zr_6_(O)_4_(OH)_4_]^12+^ clusters connected by polycarboxylato ligands [3,4]. Gels based on Zr(IV) and polycarboxylate ligands [5,6] are complex materials that combine the properties of polymers with the capabilities provided by the presence of zirconium atoms to generate an uncountable number of applications, such as sensors [7], energy storage [8], gas storage/removal [9], etc.

**Figure 1:**
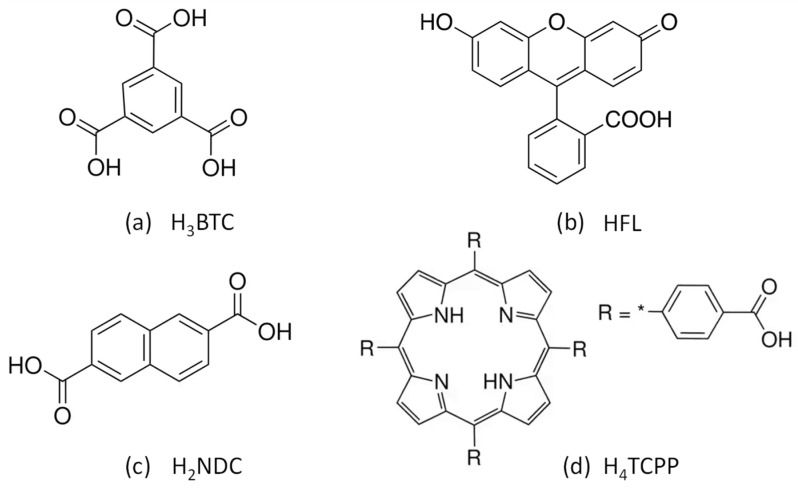
Bridging ligand and selected fluorophore molecules: (**a**) benzene-1,3,5-tricarboxylic acid, (**b**) fluorescein, (**c**) naphthalene-2,6-dicarboxylic acid, and (**d**) 4,4′,4″,4‴-(porphine-5,10,15,20-tetrayl)tetrakisbenzoic acid.

Most of the MOGs reported are obtained through an energetically demanding route (i.e., employing heating procedures) and with environmentally unsustainable solvents such as N,N-dimethylformamide, N,N-dimethylacetamide, or hydrochloric acid [6,10]. In our research group, we have prepared several zirconium-based metal–organic gels containing the abovementioned [Zr_6_(O)_4_(OH)_4_]^12+^ SBU (Secondary Building Unit) and polycarboxylic ligands using a rapid method (1–2 min) at room temperature with green solvents and without employing modulators such as HCl and acetic acid [10]. Among them, a thermally and chemically robust MOG of general formula [Zr_6_(O)_4_(OH)_4_(BTC)_2.13_(HBTC)_2.81_]·n solvent was synthesized, where BTC is the benzene-1,3,5-tricarboxylate anion (trimesate anion). The porosity and features of this material allowed us to use it as a stable catalyst in the continuous electroreduction of CO_2_ [11]. The aim of the present work was to take advantage of the characteristics of this material and introduce different molecules with luminescent properties [12] into its structure so that it could be used as a sensor [13,14]. Three fluorophores were selected (Figure 1): fluorescein (HFL), which contains a single carboxylic group capable of bonding with Zr metal atoms; naphthalene-2,6-dicarboxylic acid (H_2_NDC) [15], which has two carboxylic groups; and 4,4′,4″,4‴-(porphine-5,10,15,20-tetrayl)tetrakisbenzoic acid (H_4_TCPP) [13,16,17], a molecule with four bridging-capable carboxylic groups. These molecules show a luminescent response in blue, yellow, and red, respectively, which is retained when they are anchored to the Zr(IV) metal centers. In principle, these fluorophores in their anionic form will replace some of the trimesate anions in the gel. Interestingly, the luminescence emission will undergo significative changes when this functionalized MOG is suspended in different solvents or in aqueous solutions containing different analytes. The change in the luminescent signal of each fluorophore under these conditions can be employed for sensing purposes [18], but these changes are more specific when the three fluorophore molecules are present simultaneously in the MOG. The interaction of the adsorbed molecules modifies the luminescent signals of each fluorophore present in the MOG to a different extent to provide a characteristic color for each analyte, resulting from the sum of the three emissions. However, the emission intensity is also affected by self-quenching, and changes in the fluorophore density can generate significant variations in the emission features that hinder their use as chemical sensors.

In the following sections, we will show that the synthesis conditions that are optimal for fast gelation are also suitable for anchoring multiple carboxylic ligands, such as the fluorophore molecules described above. The resulting gels incorporating the three fluorophores provided fluorescence emissions that changed in the presence of different solvents or molecules dissolved in water. However, quantitative measurements were not possible because the emission signals were not reproducible due to the poor mechanical properties of the resulting gels. Finally, we provide a successful approach to overcome this drawback by creating a composite material [19] between the fluorescent MOG and the micrometric quartz particles that allow reproducible quantitative measurements. The latter, together with an innovative sample preparation method that allows the fluorescence emission measurement to be carried out while the particles are immersed in the liquid, allows the development of a method for the unambiguous identification of different solvents and analytes in aqueous solutions by displaying the relative intensities of the three fluorophore signals in a 3D plot.

## 2. Results and Discussion

**Figure 2:**
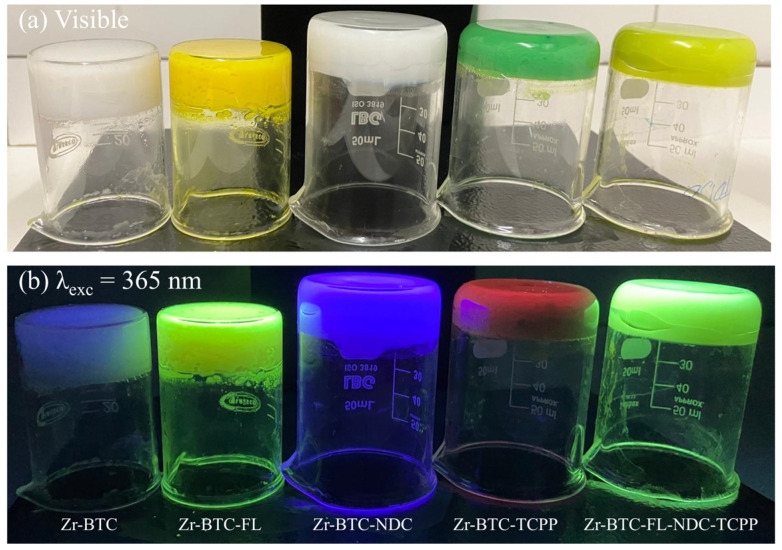
Freshly prepared MOGs under visible light (**a**) and under UV excitation (**b**). Note that after washing the gel with methanol over 48 h and exchanging the solvent four times per day, the luminescence of Zr-BTC-FL turned yellow and that of Zr-BTC-NDC-FL-TCPP turned more whitish.

**Figure 3:**
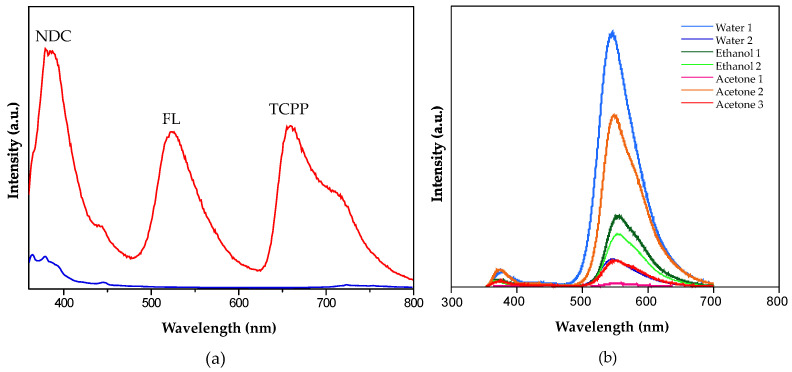
(**a**) Fluorescence intensity of Zr-BTC-NDC-FL-TCPP (red) and Zr-BTC (blue) gels excited at 325 nm. (**b**) Intensity of the fluorescent signals of the monoliths of the Zr-BTC-FL sample in different solvents, where the poor reproducibility of the measurements becomes evident.

To develop a fluorescent sensor based on these materials, three fluorophores whose fluorescence emission closely matches the three primary colors (blue, yellow, and red) were selected: naphthalene-2,6-dicarboxylic acid (H_2_NDC), fluorescein (HFL), and 4,4′,4″,4‴-(porphine-5,10,15,20-tetrayl)tetrakisbenzoic acid (H_4_TCPP). Their combination within the same porous matrix provides a material that, upon excitation with the same wavelength, changes the emission of each fluorophore in a non-equal way, resulting in color changes that are visible to the naked eye. The fast gelation conditions obtained under the above-described synthetic conditions facilitates the incorporation of these complex mixtures of fluorophores by the coordination of their deprotonated counterparts to the zirconium metal centers, avoiding any possible segregation that could take place under slower thermodynamically driven reaction conditions. As result, this approach provided MOGs with deep blue (421 nm; Zr-BTC-NDC), yellow (530 nm; Zr-BTC-FL), and red (680/712 nm; Zr-BTC-TCPP) emissions (Figure 2 and Figure 3a). It is worth noting that the Zr-BTC gel also produces a fluorescence response in the violet spectrum (393 nm) but it is too weak to be used effectively for sensing purposes. The fluorescence maximum of the fluorophores anchored to the Zr-BTC system undergoes a slight redshift compared to the free molecules dissolved in water (380 nm for H_3_BTC [20,21], 426 nm for H_2_NDC [22,23], 500 nm for HFL [24], and 643/706 nm for the Q(0,0) and Q(0,1) emissions of H_4_TCPP [25,26,27]).

**Figure 4:**
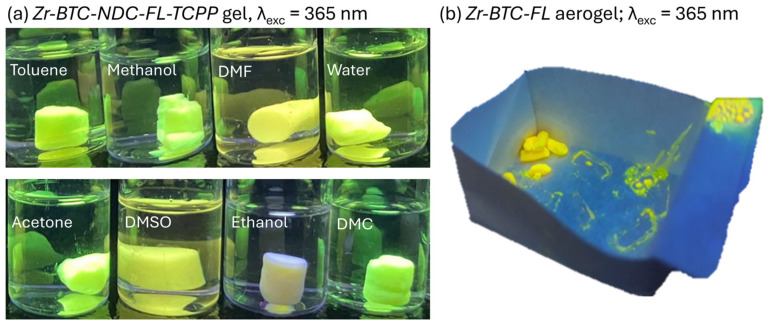
(**a**) Fluorescence emissions of freshly prepared Zr-BTC-NDC-FL-TCPP MOGs under 365 nm excitation when immersed in different solvents. (**b**) Fluorescence emissions under 365 nm excitation of the Zr-BTC-FL aerogel.

Upon exposure of the three fluorophores incorporated into the Zr-BTC-NDC-FL-TCPP gel to different solvents, visual inspection of the samples revealed changes in the fluorescence emissions (Figure 4a). However, when quantitative measurements were attempted, there was no reproducibility. This is because any manipulation of the gel, such as that required to place the sample in the fluorimeter, leads to deformations/densifications of the monolith that also affect the intensity of the fluorescence emissions due to self-quenching effects (Figure 3b). The corresponding aerogel was also prepared by CO_2_ supercritical drying and it retained the fluorescence (Figure 4b). However, the resulting monoliths were so fragile, that again, there was no way to obtain reproducible luminescence measurements.

**Figure 5:**
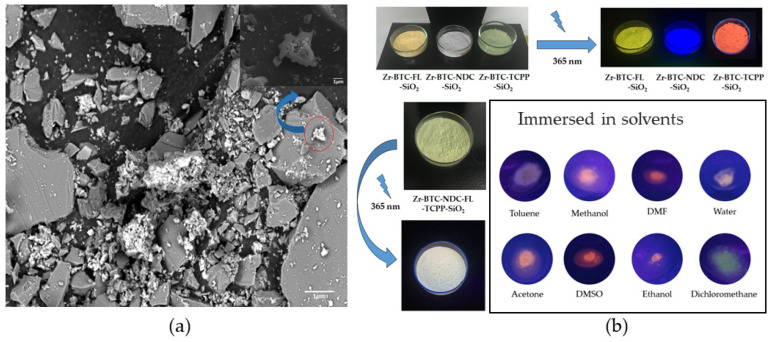
(**a**) SEM micrographs at 1000× magnification of Zr-BTC-FL-SiO_2_ as a representative case of MOG-SiO_2_ composites. Upper right inset: selected area at 25,000× magnification showing details of the MOG. (**b**) Images of the MOG-SiO_2_ composites. Top left: color of the composites containing a single fluorophore irradiated by daylight. Top right: fluorescence emission of the composites containing a single fluorophore irradiated at 365 nm. Bottom left: Zr-BTC-NDC-FL-TCPP-SiO_2_ under daylight and irradiated at 365 nm (notice the white color emerging from the combination of the three elemental colors). Bottom right: colors emerging from the combination of the fluorescence emissions of Zr-BTC-NDC-FL-TCPP-SiO_2_ when immersed in different solvents and excited at 325 nm.

**Figure 6:**
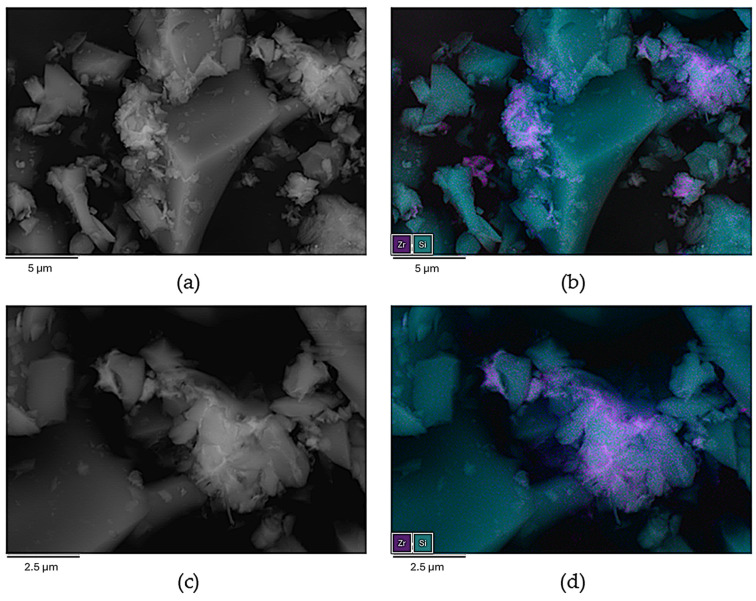
(**a**,**c**) SEM backscattered electron images of Zr-BTC-NDC-FL-TCPP-SiO_2_ and (**b**,**d**) projected map of elemental distribution.

**Figure 7:**
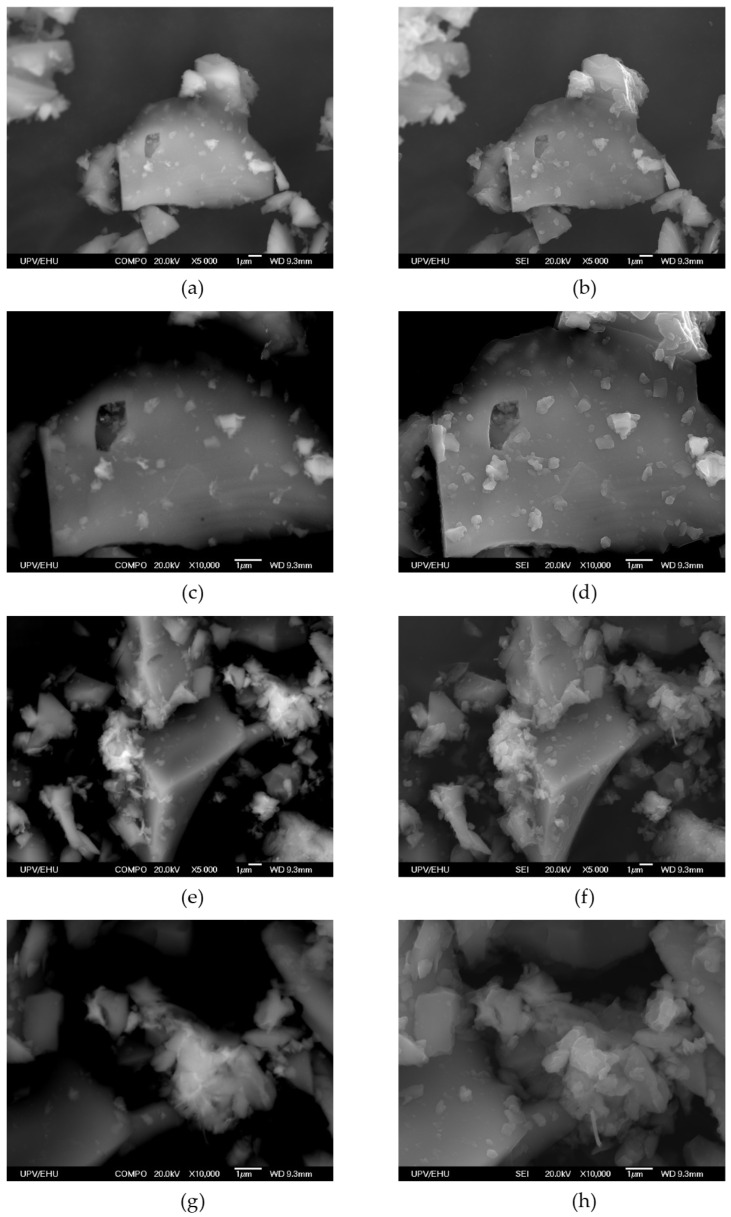
(**a**,**c**,**e**,**g**) SEM backscattered electron images of Zr-BTC-NDC-FL-TCPP-SiO_2_ and (**b**,**d**,**f**,**h**) SEM secondary electron images.

Therefore, an alternative strategy was approached in which, instead of generating macroscopic gel monoliths, nanometric particles of the gel were grown within a matrix of SiO_2_ microcrystals intended to protect the fluorescent metal–organic nanoparticles from external mechanical stress. SiO_2_ (quartz) was selected because of its transparency toward visible light and a significant portion of UV light (>200 nm). The preparation of this mixture was performed using the same conditions that were used for metallogels, but micrometric quartz particles (90% quartz and 10% metallogel) were added under vigorous stirring conditions for 12 h. The resulting product was thoroughly washed with methanol using a soxhlet apparatus until the washing liquid remained colorless and did not show any fluorescence emission upon exposure to a UV lamp (365 and 254 nm). Finally, the product was dried at room temperature. SEM/EDX images showed that the SiO_2_ microcrystals were surrounded by far smaller agglomerates of particles of the Zr-BTC-NDC-FL-TCPP gel (Figure 5a, Figure 6 and Figure 7). Under these conditions, the fluorescence response remained stable during manipulation, enabling quantitative measurements. The big size difference between the quartz microcrystals and the MOG nanoparticles prevents the compaction of these particles when pressure is applied. The packing of the quartz microparticles, even under pressure, creates voids that allow the far smaller MOG particles to accumulate within them and withstand the pressure without affecting the fluorescence emission. In addition, the composite material exhibits chemical stability comparable to that of the pristine (non-silica containing) metallogel. The preliminary visual inspection of the single component MOG-SiO_2_ composites provided the expected red, green, and blue emissions for Zr-BTC-TCPP-SiO_2_, Zr-BTC-FL-SiO_2_, and Zr-BTC-NDC-SiO_2_, respectively (Figure 5b). Since MOG-SiO_2_ composites exhibited a reproducible fluorescence response, a fine-tuning of the fluorophore concentrations in Zr-BTC-NDC-FL-TCPP-SiO_2_ was performed until the combination of the three emissions provided a white emission (Figure 5b). The best results were obtained using 1.66 mmol ZrCl_4_, 2.716 mmol H_3_BTC, 0.030 mmol HFL, 0.282 mmol H_2_NDC, and a surprisingly low value of only 2.5 × 10^−3^ mmol H_4_TCPP due to its very effective self-quenching capacity. The immersion of the composites in different solvents produced fluorescence emission color changes related to the non-equivalent changes in the response of each fluorophore; these changes were appreciable visually (Figure 5b) and registered in the spectra discussed below. The possible leaching of the active fluorescence molecules and zirconium atoms when exposed to these solvents and to different molecules dissolved in water was checked by fluorescence analysis and elemental analysis of the washing liquid. The results showed that leaching was negligible, and that the anchorage of the active molecules was strong enough for their involvement in sensing applications. Emission color changes were also observed in the presence of different organic molecules (benzyl alcohol, caffeine, fructose, glucose, imidazole, phenol, and urea) dissolved in water when the samples were immersed in these solutions. Despite the fact that the MOG-SiO_2_ mostly remained stable, thereby allowing its reutilization, it was noticed that carboxylic molecules, or other molecules with functional groups that can strongly coordinate to the zirconium metal center, create a significant leaching of the fluorophore molecules (mainly fluorescein as deduced from the resulting green/yellow fluorescence observed in the mother liquid).

**Figure 8:**
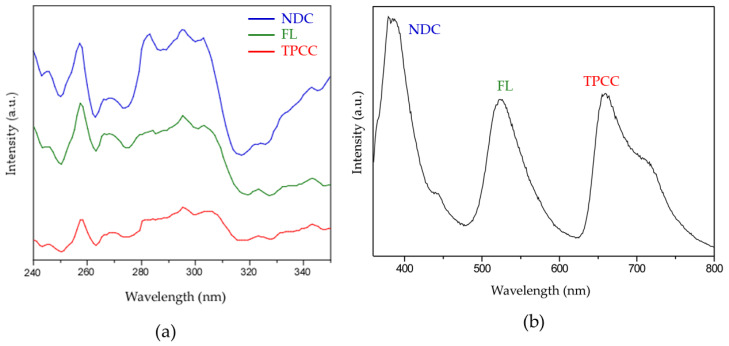
(**a**) Excitation spectra of the three fluorophores employed. (**b**) Emission spectra of Zr-BTC-NDC-FL-TCPP-SiO_2_ excited at 325 nm.

The three emission maxima present in the multifluorophore Zr-BTC-NDC-FL-TCPP-SiO_2_ sample provided the opportunity to use their fluorescence spectra for the univocal identification of chemical species based on a stimuli–response interaction with the MOG matrix. The eye sensitivity is greater for green and lower for blue and red colors [28]. This means that although we perceive a white emission through our eyes, the intensity of the emission from the three fluorophores may not be equal. However, as our goal was to develop a quantitative sensoric system, we tried to get these emission intensities as close as possible to achieve similar sensitivity for each fluorophore. To achieve this, the excitation spectrum for each fluorophore was measured and the excitation wavelength was fixed at 325 nm, the value in which three fluorophore emissions are closer (Figure 8).

**Figure 9:**
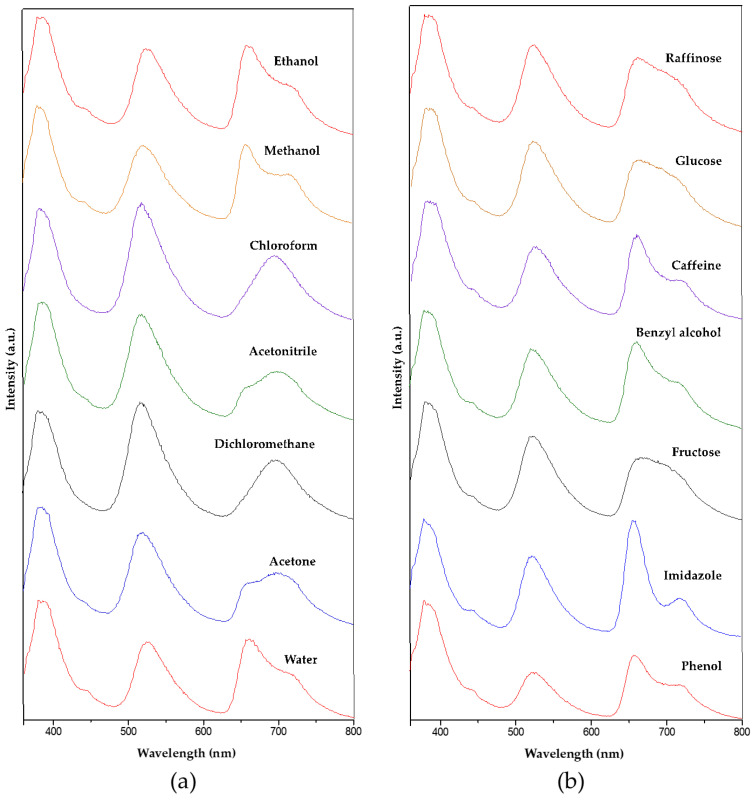
Fluorescence emissions of Zr-BTC-NDC-FL-TCPP-SiO_2_ when immersed in different solvents (**a**) and molecules dissolved in water (0.1 g/mL) (**b**).

**Figure 10:**
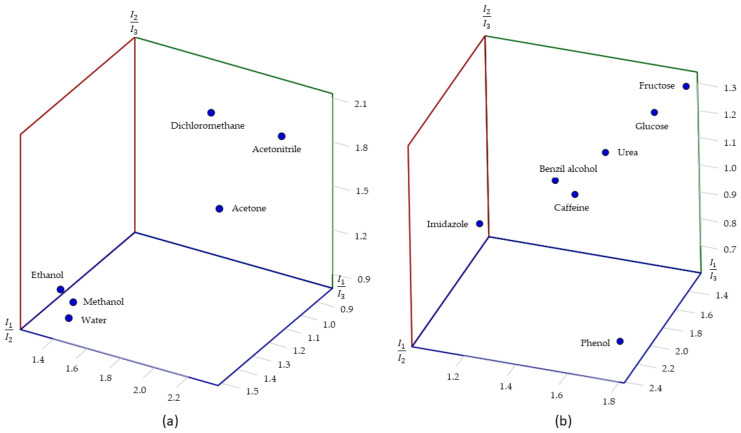
Representation of the relative intensity fluorescence emission values in a three-dimensional map for the solvents (**a**) and molecules dissolved in water (**b**). I_1_, I_2_, and I_3_ correspond to the intensity at the maximum of the peak corresponding to NDC, FL, and TCPP fluorophores, respectively.

The quantitative analysis of intensity of each fluorescence emission could in principle provide a sensing opportunity for the solvents and for the analytes dissolved in water if the mass concentration of the analyte is kept fixed (0.1 mg/mL). However, as the fluorescence signal’s intensity depends greatly on the excitation intensity of the UV source and the amount of the sample, among other parameters, a different approach was used that employed relative intensities. As we had three fluorophores incorporated simultaneously, their main emissions NDC (I_1_; 387 nm), FL (I_2_; 525 nm), and TCPP (I_3_; 690 nm) and their three relative intensity values I_1_/I_2_, I_1_/I_3_, and I_2_/I_3_ were employed for the analysis. These measurements (Figure 9) must be performed while the solid sample is immersed in the solvent or aqueous solution, which implies a specific setup for the experiment. A quartz cuvette that was originally designed for liquid measurement was used. The Zr-BTC-NDC-FL-TCPP-SiO_2_ composite (0.1 g) immersed in the solvent or aqueous solution (10 mL) was transferred to this cuvette. The particles were forced to deposit on the quartz window using a centrifuge. The whole procedure was repeated three times for every solvent or analyte to verify the reproducibility of the data (Table 1 and Table 2). A detailed description of the procedure can be found in the Materials and Methods section and in the video provided in the Supplementary Materials section. By depicting these relative intensity values in a 3D diagram, the solvents and analytes could be easily distinguished (Figure 10).

A comparable method could be developed using samples that contain a single fluorophore [7,28,29], but the results for different solvents/molecules would spread in just one dimension and provide less differentiated results or less specificity in the identification. Another option would be to employ three sets of samples with just one fluorophore in each one, but strict control of the sample amount and measurement setup would be required.

## 3. Conclusions

Zirconium polycarboxylate gels functionalized with multiple fluorophores were successfully developed for advanced fluorescence sensing applications. By incorporating fluorescein, naphthalene-2,6-dicarboxylic acid, and 4,4′,4″,4‴-(porphine-5,10,15,20-tetrayl)tetrakisbenzoic acid into a robust zirconium-based gel matrix, the material demonstrated selective and tunable fluorescence responses to different solvents and analytes. The incorporation of quartz microcrystals improved mechanical stability, enabling reproducible fluorescence measurements. The ability of the composite to detect and differentiate analytes in aqueous solutions highlights its potential for chemical sensing applications [30,31,32].

This approach of creating mechanically robust fluorescent MOG-SiO_2_ composites can be extrapolated to some other fluorophore molecules and metal–organic materials, providing a great source of sensing materials. Additionally, it would be interesting for future research to investigate how the fluorescence emissions change in the presence of solvent mixtures or when two molecules are in solution, and if there is a way to correlate these changes with the composition of the liquid media. Results of the latter would open the door to its application as a sensor in more complex systems, such as those needed for environmental monitoring or biomedical diagnostics.

## 4. Materials and Methods

Zirconium(IV) chloride, benzene-1,3,5-tricarboxylic acid (trimesic acid, H_3_BTC), fluorescent molecules, and solvents were purchased from Sigma-Aldrich (St Louis, MO, USA) and used without any prior purification. Additionally, silicon dioxide particles (Sigma-Aldrich: sand, white quartz powder, >230 mesh) were employed for the formation of the gel–quartz composites.

### 4.1. Synthesis of MOGs and MOG-SiO_2_ Composites Functionalized with Fluorophore Molecules

Following a previously described procedure [9], ZrCl_4_ (0.389 g, 1.66 mmol) dissolved in a methanolic/aqueous solution (4.8 mL and 0.2 mL, respectively) was added to trimesic acid (0.231 g, 1.11 mmol) dissolved in 8 mL methanol, resulting in a Zr:COOH molar ratio of 1:2, as present in the [Zr_6_O_4_(OH)_4_(OOCR)_12_] SBU [33]. The final solution was sonicated on an ultrasound bath (P selecta Ultrasons-H) at room temperature until the solution turned from transparent to translucent (approx. 1–2 min). The vial inversion probe corroborated the correct gelation of the reaction mixture. In the case of the MOG compounds with the fluorescence molecules, the corresponding fluorophore molecule was added in the trimesic acid solution, maintaining the Zr:COOH group’s molar ratio of 1:2 (Table 3). The same conditions were employed to obtain the MOG-SiO_2_ composites; however, micrometic powder quartz crystals (10 g, 170 mmol) were added and vigorous stirring was applied for 24 h instead of using sonication. The obtained samples were thoroughly washed in water for 12 h (replacing the water every 2 h) to remove all the remnants of the unreacted reagents. Later, the sample was collected by filtration, washed with ethanol, and dried under room temperature conditions. Samples were stored so that they were protected from the light.

### 4.2. Fluorescence Measurements

The fluorescence measurements were performed on an Agilent Technologies Cary eclipse Fluorescence Spectrophotometer. The MOG measurements were performed by placing the gels in sample holders for solids in which the sample is held between a high-performance Quartz Glass and a presser. The observed fluorescence highly depends on the amount of pressure applied. The measurements on the composite Zr-BTC-NDC-FL-TCPP-SiO_2_ were completed using Hellma^®^ (Müllheim, Germany) micro absorption cuvettes (High Performance Quartz Glass, spectral range 200–2500 nm, pathlength 10 mm, chamber volume 700 μL). The measurement of the fluorescence emission while immersed in the different solvents and aqueous solutions was accomplished using the following procedure. A total of 0.1 g of Zr-BTC-NDC-FL-TCPP-SiO_2_ and 10 mL of the corresponding solvent or aqueous solution were placed in a test tube and left for 20 min with gentle stirring. Later, the test tube was centrifugated at 3000 rpm for 5 min on a LAN.TECHNICS (INILAB, Madrid, Spain) centrifuge. The solid was then transferred to the Hellma^®^ micro absorption cuvette using a Pasteur pipette, ensuring that the composite particles were always immersed in the liquid by filling the curvette with these solvents or aqueous solutions. The cuvette was then placed in the centrifuge with its glass window aligned with the rotation axis. The centrifuge force made the particles of the composite–sample pile up in the outer quartz window. The cuvette was then placed in the fluorescence spectrometer and the measurement was taken. The whole procedure was repeated three times for every reported data element to verify the reproducibility of the measurements (Table 1 and Table 2). The entire measuring procedure is detailed in a video provided in the Supplementary Materials section.

### 4.3. Physical Measurements

Scanning electron microscopy (SEM) measurements of the samples were conducted on an FEG-SEM JEOL 7000F (JEOL, Tokyo, Japan) scanning electron microscope in secondary electron (SE) and backscattered electron (BSE) modes; at magnifications between ×1k and ×10k; and using an accelerating voltage of 20 kV, a current intensity of 1 nA, and an approximate working distance of 10 mm. Elemental mapping during SEM analysis was performed by energy-dispersive X-ray spectroscopy (EDX) using an Oxford INCA X-sight Series Si(Li) pentaFET detector. Prior to SEM measurements, the samples were attached to the sample holder using double-sided adhesive carbon tape and coated with a carbon layer by sputtering using the Q150T sample preparation kit (Quorum Technologies Ltd., Laughton, UK). Fourier-transform infrared (FTIR, KBr pellets) spectra of the samples were recorded at a resolution of 4 cm^−1^ in the 4000–400 cm^−1^ region using a FTIR 8400S Shimadzu spectrometer. The KBr pellets of the compounds were prepared at a concentration of 2–3% using spectroscopic grade KBr (Sigma-Aldrich) that was previously dried at 130 °C. Thermal analysis (TGA) was performed on a METTLER TOLEDO (Greifensee, Switzerland) TGA/SDTA851 thermal analyzer in a synthetic air (80% N_2_, 20% O_2_) flux of 50 cm^3^/min, from room temperature to 800 °C with a heating rate of 5 °C/min, using alumina crucibles, and using a sample size of about 10–20 mg per run.

## Figures and Tables

**Table 1:** Mean value, standard deviation, and ratios of the fluorescence intensities (a.u.) for Zr-BTC-NDC-FL-TCPP-SiO_2_ when immersed in different solvents and aqueous solutions of different molecules (0.1 g/mL).

**Solvents**	**I_1_** **(NDC)**	**I_2_** **(FL)**	**I_3_** **(TCPP)**	**I_1_/I_2_**	**I_1_/I_3_**	**I_2_/I_3_**
Water	513 (2)	337 (4)	344 (10)	1.52 (2)	1.49 (4)	0.97 (3)
Acetone	553 (9)	437 (4)	257 (6)	1.27 (2)	2.15 (6)	1.70 (4)
Methanol	624 (4)	431 (12)	431 (21)	1.45 (4)	1.45 (7)	1.00 (6)
Ethanol	511 (4)	377 (10)	396 (14)	1.36 (4)	1.29 (5)	0.95 (4)
Acetonitrile	583 (13)	521 (2)	245 (6)	1.12 (3)	2.38 (8)	2.13 (5)
Dichloromethane	405 (5)	437 (12)	228 (2)	0.93 (3)	1.78 (1)	1.92 (3)
**Aqueous Solutions**	**I_1_** **(NDC)**	**I_2_** **(FL)**	**I_3_** **(TCPP)**	**I_1_/I_2_**	**I_1_/I_3_**	**I_2_/I_3_**
Glucose	514 (8)	372 (13)	302 (13)	1.38 (4)	1.70 (8)	1.23 (6)
Urea	520 (2)	364 (7)	343 (22)	1.43 (3)	1.52 (10)	1.06 (7)
Fructose	516 (6)	379 (8)	283 (7)	1.36 (3)	1.82 (5)	1.34 (4)
Imidazole	521 (7)	369 (9)	512 (14)	1.41 (4)	1.02 (3)	0.72 (3)
Benzyl alcohol	507 (5)	358 (9)	385 (9)	1.42 (4)	1.32 (3)	0.93 (3)
Caffeine	477 (9)	314 (27)	337 (9)	1.52 (14)	1.42 (17)	0.93 (14)
Phenol	434 (6)	180 (2)	239 (2)	2.41 (4)	1.81 (3)	0.75 (1)

**Table 2:** Fluorescence intensity values (a.u.) for Zr-BTC-NDC-FL-TCPP-SiO_2_ when immersed in different solvents and aqueous solutions of different molecules (0.1 g/mL). Three independent measurement values are reported.

Solvents	I_1_ (NDC)	I_2_ (FL)	I_3_ (TCPP)	Aqueous Solutions	I_1_ (NDC)	I_2_ (FL)	I_3_ (TCPP)
Water	513	336	333	Glucose	505	368	314
	515	341	353		516	368	289
	511	334	347		521	381	304
Acetone	562	433	251	Urea	518	356	328
	553	440	257		521	368	332
	545	439	262		520	369	368
Methanol	622	443	411	Fructose	511	380	276
	622	420	428		522	386	289
	629	429	453		514	371	283
Ethanol	512	381	393	Imidazole	517	369	510
	514	365	384		529	378	499
	506	384	412		518	361	526
Acetonitrile	598	520	240	Benzyl alcohol	508	349	379
	576	523	251		512	366	381
	574	521	245		502	359	395
Dichloromethane	411	451	229	Caffeine	486	346	383
	402	432	226		468	297	311
	402	429	228		477	300	316
				Phenol	427	182	241
					438	179	237
					436	178	239

**Table 3:** Sample coding and reagent amounts employed for the synthesis of MOGs.

Code	H_3_BTC (mmol)	HFL (mmol)	H_2_NDC (mmol)	H_4_TCPP (mmol)
Zr-BTC-FL	1.090	0.050	-	-
Zr-BTC-NDC	0.560	-	0.820	-
Zr-BTC-TCPP	1.105	-	-	0.001
Zr-BTC-NDC-FL-TCPP	1.069	0.050	0.030	0.001

## Data Availability

The original contributions presented in the study are included in the article/Supplementary Materials, further inquiries can be directed to the corresponding authors.

## Supplementary Materials

The following supporting information can be downloaded at https://www.mdpi.com/article/10.3390/gels10120783/s1, Figure S1: FT-IR spectra of the MOG-SiO_2_. Figure S2: Thermogravimetric analysis of Zr-BTC-F/NDC/TCPP-SiO_2_ performed under a synthetic air (80% N_2_/20% O_2_) atmosphere with a heating rate of 10 °C/min. Video S1: The entire Zr-BTC-F/NDC/TCPP-SiO_2_ fluorescence measuring procedure.
