# Supplementary material for: Zirconium–Polycarboxylato Gel Systems as Substrates to Develop Advanced Fluorescence Sensing Devices
Source: Gels. 2024 Nov 29;10(12):783. doi: 10.3390/gels10120783 (PMC11675840; doi:10.3390/gels10120783)
Supplement: Supplementary file 1 [file gels-10-00783-s001.zip › gels-3304619-supplementary/gels-3304619-supplementary-v4.pdf]

Zirconium-Polycarboxylato Gel Systems as Substrate to Develop Advanced Fluorescence Sensing Devices

Jon Pascual Colino [1,2*], Garikoitz Beobide [1,2], Oscar Castillo [1,2*], Javier Cepeda [3], Mónica Lanchas [4], Antonio Luque [1,2] and Sonia Pérez-Yáñez[1,2]

[1] Departamento de Química Orgánica e Inorgánica, Facultad de Ciencia y Tecnología, Universidad del País Vasco, UPV/EHU, Apartado 644, E-48080 Bilbao, Spain
[2] BCMaterials, Basque Center for Materials, Applications and Nanostructures, UPV/EHU Science Park, E-48940 Leioa, Spain
[3] Departamento de Química Aplicada, Facultad de Química, Universidad del País Vasco, UPV/EHU, 20018 Donostia-San Sebastián, Spain
[4] Departamento de Ingeniería Química, Facultad de Ciencia y Tecnología, Universidad del País Vasco, UPV/EHU, Apartado 644, E-48080 Bilbao, Spain
* Correspondence: JPC: jon.pascual@ehu.eus; OC: oscar.castillo@ehu.eus

INDEX

1.- FT-IR SPECTROSCOPY 3

2.- THERMOGRAVIMETRY 4

1.- FT-IR SPECTROSCOPY

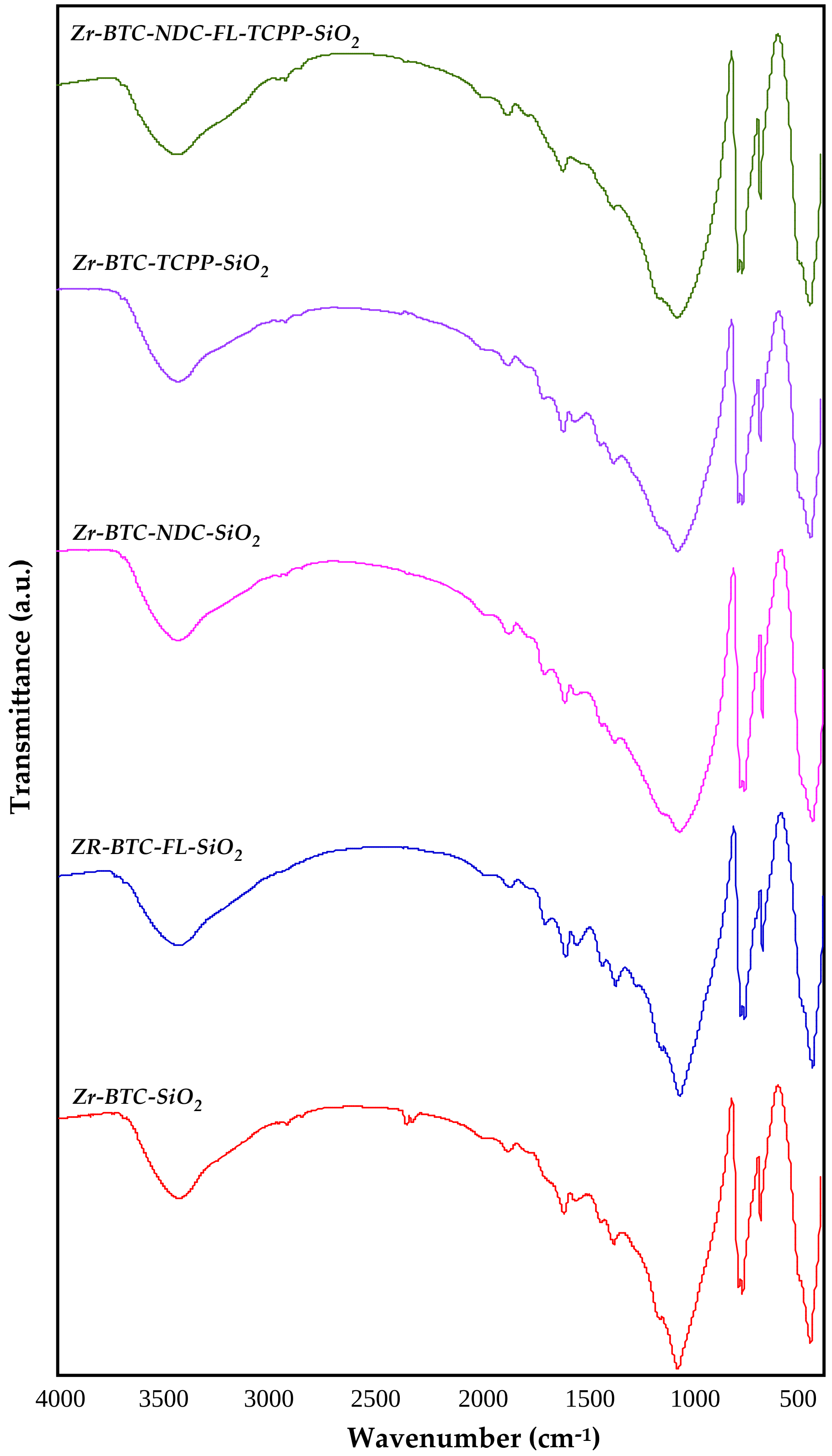

Figure S1. FT-IR spectra of the MOG-SiO_2 samples.

2.- THERMOGRAVIMETRY

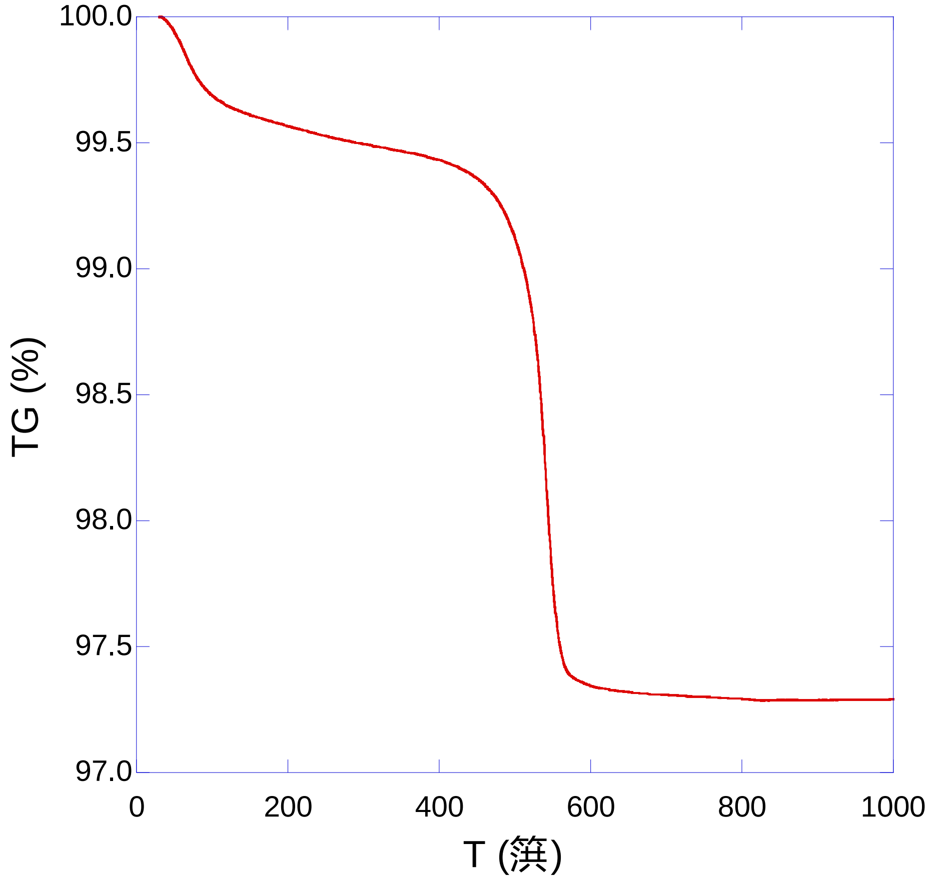

Figure S2. Thermogravimetric analysis of *Zr-BTC-F/NDC/TCPP-*SiO_2 performed under a synthetic air (80% N_2/20% O_2) atmosphere with a heating rate of 10 °C/min.
